# Iron‐Sulfur Cluster Targeting: A Novel Perspective for Treating Alcohol‐Related Lipid Metabolic Diseases

**DOI:** 10.1002/jbt.71082

**Published:** 2026-08-17

**Authors:** Li Xu, Zhenyang Xu, Chenli Su, Yanqian Zhao, Linjing Qiang, Xiayong Jing, Yuxiang Sun

**Affiliations:** ^1^ School of Basic Medical Sciences & School of Public Health, Faculty of Medicine, Yangzhou University Yangzhou PR China; ^2^ Key Laboratory of the Jiangsu Higher Education Institutions for Integrated Traditional Chinese and Western Medicine in Senile Diseases Control (Yangzhou University) Yangzhou PR China; ^3^ Hangzhou Tangji Medical Technology Co. Ltd. Hangzhou PR China; ^4^ Shaanxi University of Chinese Medicine PR China; ^5^ Department of Urology Affiliated Hospital of Yangzhou University Yangzhou PR China

**Keywords:** alcohol, iron–sulfur cluster, liver, mitochondria, redox

## Abstract

Iron–sulfur (Fe–S) clusters are pivotal molecular cofactors bridging inorganic chemistry and organic life processes, with their tightly regulated biosynthesis and function underpinning cellular metabolic homeostasis. Chronic alcohol‐induced metabolic stress impairs Fe–S cluster stability and biosynthesis via reactive oxygen species (ROS)‐mediated oxidative damage and direct acetaldehyde toxicity, culminating in functional failure of this core cofactor. Current evidence is synthesized herein to delineate the hierarchical pathogenic cascade triggered by Fe–S cluster dysfunction. At the cellular level, Fe–S cluster depletion inactivates aconitase, driving citrate accumulation and redirecting carbon flux toward lipogenesis; concurrently, succinate dehydrogenase dysfunction causes succinate buildup, which epigenetically suppresses fatty acid oxidation. Impaired tRNA thiolation further compromises mitochondrial translational fidelity. Together, these defects precipitate an energy crisis and a self‐perpetuating lipotoxicity–oxidative stress vicious cycle. At the systemic level, this metabolic dysregulation fuels disease progression from fatty liver and hyperlipidemia to atherosclerosis, mediated by enhanced hepatic VLDL secretion, reduced HDL levels, and oxidized LDL (Ox‐LDL) formation. Furthermore, a hypothetical structural model for acetaldehyde‐driven Fe–S cluster disintegration, grounded in organometallic chemistry principles. Collectively, this review highlights the central role of Fe–S clusters in alcohol‐associated metabolic disorders and provides a novel theoretical framework for developing therapeutics targeting Fe–S cluster homeostasis.

## Graphical abstract

Alcohol metabolism induces structural instability of iron‐sulfur clusters through ROS‐mediated oxidative damage and direct covalent modification of acetaldehyde. The iron‐sulfur cluster deficiency drives metabolic reprogramming by inactivating aconitase, accumulating succinate, and impairing tRNA thiolation, thereby promoting hepatic steatosis, dyslipidemia, endothelial injury, and atherosclerosis progression at the systemic level.

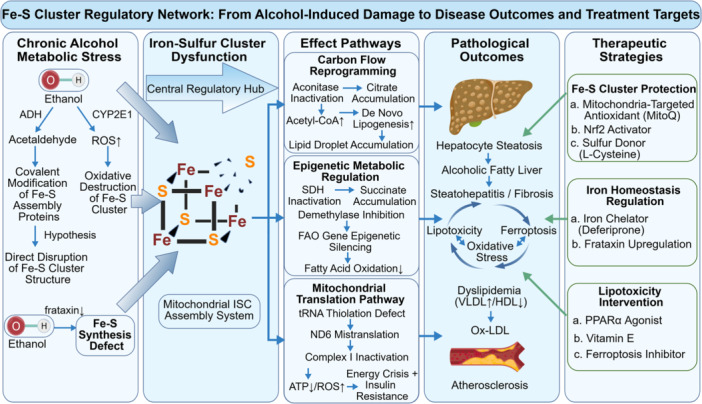

## 1 Introduction

**Figure 1:**
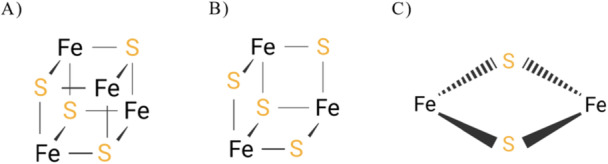
Common types of Fe–S clusters. A: [4Fe–4S], B: [3Fe–4S], C: [2Fe–2S].

Iron–sulfur (Fe–S) clusters are highly conserved inorganic cofactors in living systems, with an evolutionary origin traceable to the hydrothermal environments of primitive life, maintaining core functions across the three domains of Bacteria, Archaea, and Eukarya [1]. Through characteristic topological structures such as [4Fe‐4S], [3Fe‐4S], and [2Fe‐2S], these metal prosthetic groups are extensively involved in critical biological processes including the electron transport chain, enzyme activity regulation, gene expression control, and DNA repair/replication (Figure 1) [2, 3]. Existing research confirms that Fe–S cluster biosynthesis primarily occurs in mitochondria, and its synthetic impairment directly triggers mitochondrial dysfunction. Through complex regulatory mechanisms, Fe–S clusters establish a connection between mitochondrial function, endoplasmic reticulum stress, and lipid metabolism. Fe–S cluster dysfunction leads to lipid metabolism disorders, thereby triggering various metabolic diseases. However, there is currently no comprehensive review that systematically delineates the complete pathogenic axis from alcohol‑induced Fe–S cluster injury to lipid metabolic disorders, and the multi‑level regulatory mechanisms involved have not been fully integrated.

This review elucidates the core regulatory role of iron–sulfur (Fe–S) clusters in alcohol‐associated lipid metabolic disorders. A systematic literature search was conducted using the keywords of iron–sulfur clusters and alcohol. Based on existing studies, this paper hierarchically analyzes five core aspects: the biogenesis machinery and metabolic stress‐induced structural lability of eukaryotic Fe–S clusters; the dual damage of Fe–S cluster stability and biogenesis by alcohol metabolism‐derived reactive oxygen species (ROS) and acetaldehyde; Fe–S cluster deficiency‐mediated metabolic reprogramming characterized by altered carbon flux, epigenetically inhibited fatty acid oxidation, and impaired mitochondrial translation; Fe–S cluster dysfunction‐driven pathological progression from hepatic steatosis to atherosclerosis; and potential therapeutic strategies targeting Fe–S cluster homeostasis for alcohol‐associated metabolic diseases. Combining current organometallic chemistry evidence, this article further proposes a hypothetical model for acetaldehyde‐induced structural disruption of Fe–S clusters. This review first describes the mitochondrial ISC assembly system and the regulatory networks of Fe–S cluster biogenesis, then details the impairment of Fe–S cluster integrity via oxidative and electrophilic toxicity under chronic alcohol metabolic stress, and further elaborates three interconnected cellular mechanisms underlying Fe–S cluster defect‐induced lipid metabolic dysregulation and subsequent systemic pathological changes. Finally, it summarizes promising therapeutic strategies targeting Fe–S clusters and lipotoxicity, aiming to provide novel mechanistic insights and translational directions for the prevention and treatment of alcohol‐related lipid metabolic diseases.

### 1.1 Fe–S Cluster Assembly Systems

Fe–S cluster biosynthesis in eukaryotes is primarily mediated by the mitochondrial ISC system, and its assembly mechanism can be divided into four precisely regulated steps [4]. In the initial stage of mitochondrial Fe–S cluster biosynthesis (Step I), the Nitrogen Fixation 1 Homolog–Iron‑Sulfur Cluster Assembly Enzyme (NFS1‐ISCU) complex acts as the core catalytic unit, mediating sulfur atom transfer through its active center cofactor, pyridoxal 5'‐phosphate (PLP) [5]. PLP forms a Schiff base covalent bond with cysteine, promoting sulfur atom dissociation by polarizing the C‐S bond to generate a persulfide intermediate (‐SSH), while releasing alanine as a byproduct [6]. During this process, the Cys381 thiol group of NFS1 forms a persulfide bond (Cys381‐SSH), whereas the scaffold protein ISCU accepts the sulfur atom through its conserved Cys35/Cys61 residues, ultimately completing the de novo synthesis of the [2Fe–2S] cluster on the Isu1 scaffold protein [7].

Synergistic Regulation by the Chaperone System (Step II): Assisted by the J‐type co‐chaperone Jac1, the Hsp70 family member Ssq1 drives the dissociation of the [2Fe–2S] cluster from the ISCU scaffold dependent on ATP hydrolysis [3]. The exchange factor Mge1 maintains the catalytic cycle of Ssq1 by promoting ADP/ATP exchange [8]. The released [2Fe–2S] cluster then binds to the monothiol glutaredoxin Grx5 to form a bridged dimeric complex, laying the foundation for subsequent Fe–S cluster transport or reconstitution [9].

In Step III, the topological conversion from [2Fe–2S] to [4Fe–4S] clusters requires the participation of a second Fe–S cluster assembly center. This process is executed by the late ISC complex Isa1–Isa2–Iba57. Although independent of the early ISC mechanism, this complex still relies on the [2Fe–2S] precursor delivered by Grx5. Finally, in Step IV, specific ISC‐targeting molecules (Hsc20 and Hsp70 molecular chaperones) accurately deliver the mature [4Fe–4S] cluster to various apoproteins, completing functional assembly [10, 11].

Notably, the Fe–S cluster biogenesis network is highly vulnerable. Genetic factors causing abnormal expression of assembly proteins or oxidative stress environments (ROS accumulation) can destroy the structural stability of Fe–S clusters [12]. Recent studies have revealed that dysfunction of this system is closely related to lipid metabolism imbalance: key metabolic enzymes like Pyruvate Dehydrogenase Complex (PDHC) and Acetyl‐CoA Carboxylase (ACC) all rely on Fe–S clusters to maintain activity [13]. Experiments have confirmed that inhibiting key synthases such as NFS1 not only blocks Fe–S cluster biosynthesis but also triggers mitochondrial fatty acid metabolism disorders and lipid synthesis impairment [14, 15]. Furthermore, Fe–S clusters play a central role in redox homeostasis regulation: the superoxide response protein (SoxR) senses oxidative stress signals through its Fe–S cluster [16], the Fumarate and Nitrate Reduction regulatory protein (FNR) monitors oxygen concentration changes using its Fe–S cluster structure [17], and Iron Regulatory Proteins IRP1/IRP2 regulate the expression of iron metabolism‐related genes through their Fe–S cluster status [18]. These findings highlight the pivotal position of Fe–S clusters in linking the inorganic environment with organic life activities, and the elucidation of their assembly mechanisms provides new insights for the treatment of metabolic and degenerative diseases.

### 1.2 Collapse of Fe–S Clusters Under Chronic Alcohol Metabolic Stress

Alcohol metabolism in the body is a complex process involving enzymes like Alcohol Dehydrogenase (ADH), Aldehyde Dehydrogenase (ALDH), and Cytochrome P450 2E1 (CYP2E1) [18, 19, 20]. The inactivation of these enzymes and their products can trigger various diseases, including liver and heart diseases [21, 22, 23].

The liver is the primary organ for alcohol metabolism, with 90% of alcohol absorbed by the stomach and intestines passing to the liver. Long‐term alcohol consumption increases the risk of liver diseases, including fatty liver, hepatitis, and cirrhosis [23, 24]. Alcohol is first oxidized to acetaldehyde by ADH. Acetaldehyde is a highly reactive and cytotoxic intermediate capable of damaging tissues [25]. Subsequently, ALDH detoxifies acetaldehyde into the less toxic acetate. However, excessive alcohol intake triggers ethanol‐specific pathogenic mechanisms distinct from the common lipotoxic pathways, which mainly involve acetaldehyde, cytochrome P450 2E1 (CYP2E1), redox imbalance and alcohol metabolism processes, as well as unique mechanisms mediating hepatocellular lipotoxicity and lipid peroxidation [26]. The activity of the CYP2E1 pathway is significantly enhanced, becoming a major source of oxidative stress in hepatocytes [19, 27]. When CYP2E1 metabolizes ethanol, electrons are transferred from NADPH to the heme iron center to activate oxygen, but “uncoupling” often occurs, resulting in the production of large amounts of ROS, including hydrogen peroxide (H_2_O_2_), hydroxyl radicals, and superoxide (O_2_•^−^) [28, 29]. Notably, these ROS, especially superoxide (O_2_•^−^), have high electrostatic affinity for Fe–S clusters, initiating Fe–S cluster oxidation starting from iron release, leading to cluster instability and accelerated degradation, thereby affecting the activity of specific enzymes [30, 31, 32]. Bruska et al. conducted detailed research on ROS–Fe–S cluster interactions [33]. Although most Fe–S proteins hide vulnerable clusters within polypeptide chains, some require direct cluster participation to exert activity. These exposed clusters, mainly in the [4Fe–4S] form, are more susceptible to oxidative stress [34]. Furthermore, Fe–S clusters are core components of mitochondrial functional proteins (Aconitase, Succinate Dehydrogenase) and DNA repair enzymes; their damage further exacerbates cellular energy metabolism disorders and oxidative stress, forming a toxic cycle [30, 35, 36].

**Figure 2:**
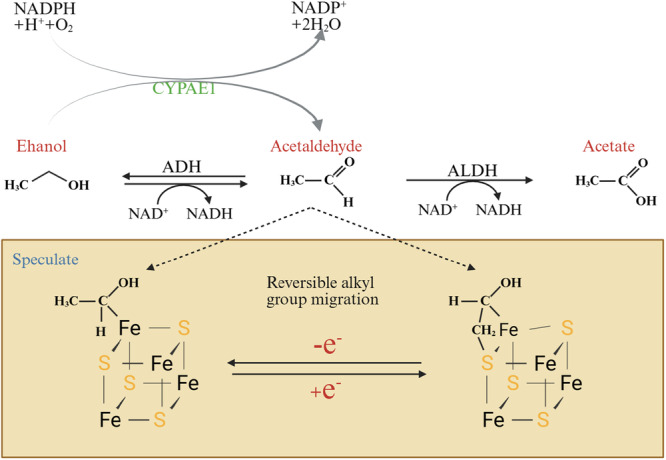
Hypothetical model of acetaldehyde‐induced damage to iron‑sulfur clusters. We hypothesize that the electrophilic carbonyl carbon of acetaldehyde may undergo nucleophilic attack by reduced iron centers on the cluster, potentially forming a transient Fe–C bond. If such an Fe–C bond were formed, subsequent alkyl migration might perturb the cluster structure; the alkyl group could migrate across the Fe–S core framework, distorting and weakening the native Fe–S bonds. This hypothetical process may alter the electronic structure and geometric configuration of the cluster, possibly leading to functional impairment or even disintegration.

Relevant studies indicate that acetaldehyde is the primary driver of alcohol‐related organ damage [19]. Acetaldehyde is highly reactive, and its electrophilic carbonyl carbon can form covalent adducts with intracellular proteins like cysteine desulfurase, scaffold proteins, and chaperone proteins, interfering with their normal functions and thereby inhibiting the effective assembly of Fe–S clusters [37, 38]. The electrophilic nature of acetaldehyde might also enable it to react directly with sulfur atoms or iron ions in Fe–S clusters, potentially leading to cluster dissociation or denaturation. This direct damage could disrupt the integrity of Fe–S clusters and may cause them to lose their function as enzyme cofactors [38]. Studies have shown that metal clusters containing sulfur atoms can undergo reversible migration of alkyl groups (‐CH_2_R) between iron and sulfur atoms [39]. Based on these observations, we hypothesize that the electrophilic carbonyl carbon of acetaldehyde might be subject to nucleophilic attack by electron‐rich sulfur anions or reduced iron centers on the cluster, putatively forming a key Fe–C bond. As a hypothetical mechanistic model, once an Fe–C bond is formed, the alkyl group might migrate, which could force a distortion of the original cubane geometry of the cluster core, potentially leading to elongation and weakening of key Fe–S bonds and disruption of the electronic structure, and ultimately possibly resulting in functional impairment or even disintegration (Figure 2).

However, the potential mechanisms of ethanol‐induced mitochondrial changes remain unclear. Notably, ethanol‐induced mitochondrial dysfunction is highly dependent on Fe–S clusters (ISCs). Additionally, direct studies have shown that ethanol inhibits frataxin expression both in vivo and in vitro. Inhibition of frataxin exacerbates ethanol‐induced ferroptosis, whereas restoration of frataxin alleviates it. Overexpression of frataxin can reverse alcohol‐induced iron metabolism disorders, enhance mitochondrial electron transport chain activity, reduce lipid peroxide levels, and significantly alleviate hepatocyte ferroptosis and liver injury, further confirming the core regulatory role of frataxin in this pathway [40].

A crucial aspect related to ethanol‐induced redox injury that warrants further consideration is its association with alterations in lipid factors, particularly those essential for cell membrane protection, for example vitamin E [26]. Ethanol exposure has been shown to interfere with vitamin E metabolism, which can significantly impair the anti‐peroxidative pathways in human hepatocytes. These pathways are vital for preventing lipotoxicity and its detrimental effects on cell membranes [41]. Such alterations can be thoroughly investigated using both targeted and untargeted lipidomic methods. Furthermore, vitamin E (alpha‐tocopherol), its phenolic terpenoid analog vitamin K, and ubiquinone are recognized as major anti‐ferroptotic agents in various tissues and specifically in liver cells, acting primarily by inhibiting phospholipid peroxidation at membranes [42, 43]. This anti‐ferroptotic role is highly relevant for mitigating alcohol‐induced lipotoxic effects. Given these insights, future research should assess the role of these and other therapeutic and hepatoprotective agents in Alcoholic Liver Disease (ALD) and discuss relevant therapeutic mechanisms and agents capable of targeting Fe–S clusters and their redox changes during ethanol exposure [44]. For instance, therapeutic strategies could involve agents that enhance vitamin E status, stabilize Fe–S clusters (e.g., iron chelators like deferiprone), provide redox buffering (e.g., N‐acetylcysteine, mito‐TEMPO), or act as direct ferroptosis inhibitors (e.g., ferrostatin‐1, liproxstatin‐1) to counteract ethanol‐induced lipotoxicity and mitochondrial collapse.

## 2 Cell Metabolic Flow Shift Driven by Fe–S Cluster Damage

### 2.1 Acute Fe–S Cluster Deficiency Leads to Carbon Flow Redirection and Lipid Droplet Generation

Aconitase is a key enzyme containing Fe–S clusters whose activity directly depends on the integrity of its Fe–S cluster structure [45]. This enzyme plays a central role in the Tricarboxylic Acid cycle (TCA), catalyzing the reversible isomerization between citrate and isocitrate [19, 46]. When Fe–S cluster deficiency causes mitochondrial or cytoplasmic aconitase to be inactivated by ROS, the isomerization process of citrate in the TCA cycle is blocked. The ratio of citrate to isocitrate changes and modulates metabolic flux within the TCA cycle, leading to intracellular citrate accumulation [47]. Research data shows that intracellular citrate levels can increase 11‐fold in Fe–S cluster‐deficient cells [47]. Citrate is known to be an allosteric inhibitor of Phosphofructokinase 1 (PFK‐1) [48]. Elevated concentrations inhibit PFK‐1, leading to glucose‐6‐phosphate accumulation in the muscles of diabetic animals, reduced glycolysis rates, and affecting fatty acid and ketone body oxidation processes in various tissues [48, 49]. This non‐hormone‐dependent acute reciprocal regulation of intracellular glucose and fatty acid oxidation by citrate is an important part of the Randles cycle regulatory pathway [50].

Furthermore, in the cytoplasm, citrate can be cleaved by ATP Citrate Lyase (ACLY) into Acetyl‐CoA and Oxaloacetate [51]. Acetyl‐CoA is a direct substrate for fatty acid and cholesterol synthesis, while oxaloacetate can participate in gluconeogenesis or re‐enter the mitochondria [52]. Therefore, citrate accumulation promotes the generation of Acetyl‐CoA in the cytoplasm, thereby driving the shift of intracellular carbon flow from energy metabolic pathways to lipid synthesis pathways [53]. These newly synthesized fatty acids combine with glycerol to form Triacylglycerols (TAG), which are stored in cells in the form of lipid droplets [19]. As dynamic organelles for storing neutral lipids within cells, the abnormal accumulation of lipid droplets is a typical marker of various metabolic diseases [51].

Crooks found that although the growth of Fe–S cluster‐deficient ISCU2^D71A^ cells is substantially inhibited, they can still perform significant glycerolipid biosynthesis [19]. This suggests that the newly synthesized fatty acids in ISCU2^D71A^ cells may not be used for new cell membrane construction but are more likely used for the formation and accumulation of lipid droplets—i.e., lipid droplets may be storage depots for de novo synthesized lipids in Fe–S cluster‐deficient cells. Further studies confirmed that citrate accumulation and increased Acetyl‐CoA production triggered by Fe–S cluster deletion directly promote fatty acid synthesis; these newly synthesized fatty acids combine with glycerol to form TAGs, which are stored in cells as lipid droplets [19]. Concurrently, related studies have also confirmed that ISCU‐mediated Fe–S cluster deficiency drives the shift of intracellular carbon flux toward fatty acid biosynthesis pathways, ultimately triggering lipid droplet accumulation [51].

**Figure 3:**
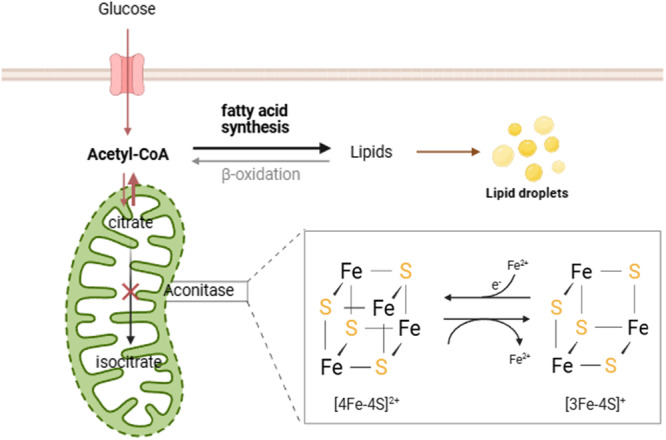
Metabolic disorders caused by failed Fe–S cluster transfer. Damage to Fe–S cluster assembly triggers significant metabolic reprogramming, particularly leading to lipid droplet accumulation in cells.

In summary, defects in Fe–S cluster biogenesis and acute iron deficiency rapidly elevate intracellular citrate concentrations, thereby inducing fatty acid synthesis and cytoplasmic lipid droplet accumulation (Figure 3). This mechanism provides an important basis for understanding the occurrence and development of related metabolic diseases like MASLD. Lipidomic characterization of hepatocyte lipotoxicity in these contexts, along with a deeper engagement with foundational Fe–S/aconitase biology, offers critical insights into the multifaceted nature of these metabolic disturbances [54]. The accumulation of ectopic fat in non‐adipose tissues, like the liver, is a central mechanism in the development of metabolic syndrome and can progress from benign steatosis to inflammatory steatohepatitis, driven by lipotoxicity. This progression involves specific remodeling of phospholipid and sphingolipid species, not just neutral lipid expansion, and correlates with early markers of lipotoxicity, including ceramide accumulation and ER stress. Therefore, while citrate‐driven de novo lipogenesis contributes to lipid droplet formation, it is part of a broader lipotoxic cascade that includes disrupted membrane homeostasis, impaired β‐oxidation, and altered iron‐regulated signaling [41, 42].

### 2.2 Oxidative Stress‐Ferroptosis Vicious Cycle Triggered by Fe–S Cluster Damage

Succinate Dehydrogenase (SDH) is a key integrative enzyme of the mitochondrial respiratory chain and the TCA cycle. Its structure consists of four subunits, all encoded by the nuclear genome: Subunit A (SDHA), which has a covalently bound Flavin Adenine Dinucleotide (FAD) cofactor, Subunit B (SDHB), which contains three Fe–S clusters and forms a soluble heterodimer serving as the catalytic center of the enzyme, and Subunits C (SDHC) and D (SDHD), which form a complete membrane region anchoring the complex to the inner mitochondrial membrane [55, 56]. The enzyme catalyzes the two‐electron oxidation of succinate to fumarate while reducing ubiquinone to ubiquinol (i.e., exerting succinate:ubiquinone oxidoreductase activity) [57]. During succinate oxidation, the two electrons it carries are first transferred to the FAD cofactor on the SDHA subunit [58], then sequentially passed through the Fe–S clusters in the SDHB subunit, and finally reach the ubiquinone binding site to complete the reduction of ubiquinone to ubiquinol [30]. Notably, the [3Fe‐4S] cluster in SDH has a low midpoint potential and is considered an energy barrier in the electron transport chain [31].

The correct assembly, maturation, and functional maintenance of SDH rely on LYR family factors (SDHAF1 and SDHAF3), which mediate the maturation process of Fe–S clusters on the SDHB subunit [57]. Defects in these assembly factors lead to impaired SDH function, thereby disrupting cellular metabolic balance [57]. Damage to Fe–S clusters themselves directly reduces the catalytic activity of SDH, decreasing succinate oxidation efficiency and ubiquinone reduction efficiency [58, 59, 60, 61]. This not only disrupts the normal operation of the TCA cycle but also damages the electron transport chain, ultimately reducing ATP production [62]. For example, when the Fe–S cluster assembly mechanism is defective in Saccharomyces cerevisiae, SDH activity is significantly impaired, triggering mitochondrial dysfunction and oxidative stress [61].

Studies have confirmed that hydrogen peroxide (H_2_O_2_) can specifically damage the binuclear Fe–S cluster N1b of respiratory chain Complex I, causing its dissociation and releasing iron ions [63, 64, 65]. For SDH, its Fe–S clusters are equally vulnerable to oxidative stress damage. Fe–S cluster damage leads to blocked electron transfer, promoting increased electron leakage, which in turn exacerbates ROS production, forming a “vicious cycle of oxidative stress‐Fe–S cluster damage‐ROS increase” [61]. Simultaneously, Fe–S cluster dissociation releases unstable free iron, expanding the intracellular “labile iron pool” [64, 66]. Excess free iron can catalyze the generation of more ROS like hydroxyl radicals, through the Fenton reaction, further aggravating oxidative damage or even triggering ferroptosis (an iron‐dependent form of cell death) [67]. In SDHB‐deficient cells, typical characteristics of iron homeostasis disorders and enhanced oxidative stress have been observed [38]. ROS attacks polyunsaturated fatty acids in cell membranes, triggering lipid peroxidation and generating harmful substances like Malondialdehyde (MDA), ultimately destroying the integrity and function of cell membranes [68]. For instance, studies on post‐harvest vibration stress in apples showed a significant correlation between SDH activity, membrane permeability, and MDA levels: ATP treatment significantly delayed the rapid decline in SDH activity caused by vibration stress, after 25 days of storage, the SDH activity in the ATP‐treated group was 39.10% higher than that in the DNP (2,4‐dinitrophenol)‐treated group, suggesting that impaired energy metabolism directly affects membrane lipid stability [68].

Furthermore, SDH is not only a metabolic enzyme, but its metabolite succinate also has important signaling molecule functions [69]. As a key intermediate product of the TCA cycle, succinate can regulate the levels of DNA and histone methylation [70]. Succinate inhibits the constitutive expression of FAO markers, including Pparα, Pgc1α, Fabp3, and Cpt2. Succinate treatment also attenuated myocardial FAO supported by palmitate, as indicated by respiratory rates (Figure 5f). All these in vitro data are similar to the in vivo observations in cs‐SDHB^−/−^ mice. The results indicate that the accumulation of succinate significantly resets the characteristics of DNA methylation and FAO gene expression in the myocardium [71].

**Figure 4:**
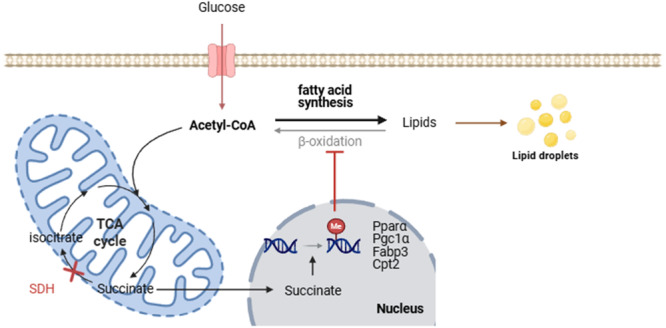
Succinate inhibits the expression of FAO genes. Deletion of SDH in the myocardium leads to succinate accumulation, thereby stimulating genome‐wide DNA hypermethylation. This epigenetic reprogramming inhibits Fatty Acid Oxidation (FAO).

In summary, the decrease or loss of SDH activity mediated by Fe–S cluster damage leads to the failure of normal succinate decomposition and subsequent intracellular accumulation. On the one hand, succinate accumulation directly blocks the TCA cycle, reducing energy supply; on the other hand, excess succinate inhibits histone demethylases, stimulates genome‐wide DNA hypermethylation [72, 73], and inhibits fatty acid oxidation (Figure 4).

### 2.3 Fe–S Cluster‐Mediated tRNA Thiolation Catalyzed by TtuA Enzyme

The coordination of Fe–S clusters in enzymes like TtuA is essential for tRNA thiolation, a modification at the wobble position (s2U34) that ensures translational fidelity and efficiency. While the biochemical transfer of sulfur via TtuA involves complex intermediate steps—including the capture of sulfur atoms and the activation of tRNA substrates—its physiological significance in the liver transcends basic sulfur metabolism [74, 75]. Emerging evidence suggests that impaired Fe–S‐dependent translational fidelity serves as a plausible mechanistic link to ethanol‐driven hepatic injury. Specifically, the 2‐thio modification of mitochondrial tRNA^Lys^ (UUU) is strictly dependent on Fe–S cluster‐containing enzymes. Deficiencies in Fe–S cluster synthesis or their disruption by ethanol‐induced oxidative stress can lead to the loss of this critical modification, triggering a cascade of mitochondrial dysfunction [76, 77]. The resulting mistranslation of mitochondrial‐encoded subunits, such as ND6, impairs the assembly of Respiratory Chain Complex I.

This disruption creates a “dual‐hit” to hepatocyte metabolism: First, Energy Crisis and Lipogenesis: A sharp decline in ATP synthesis inhibits the AMPK signaling pathway, downregulating fatty acid oxidation (e.g., CPT1A expression) and promoting lipid accumulation, a hallmark of Metabolic dysfunction‐Associated Steatotic Liver Disease (MASLD) [78, 79]. Second, Oxidative Stress and Insulin Resistance: Increased electron leakage from dysfunctional Complex I elevates ROS production [80, 81]. Excess ROS activates the JNK pathway, leading to IRS1 phosphorylation and subsequent insulin resistance. This state further exacerbates hepatic steatosis by promoting lipolysis and inhibiting lysosomal autophagy, creating a “lipotoxicity‐oxidative stress” vicious cycle [22, 82].

**Figure 5:**
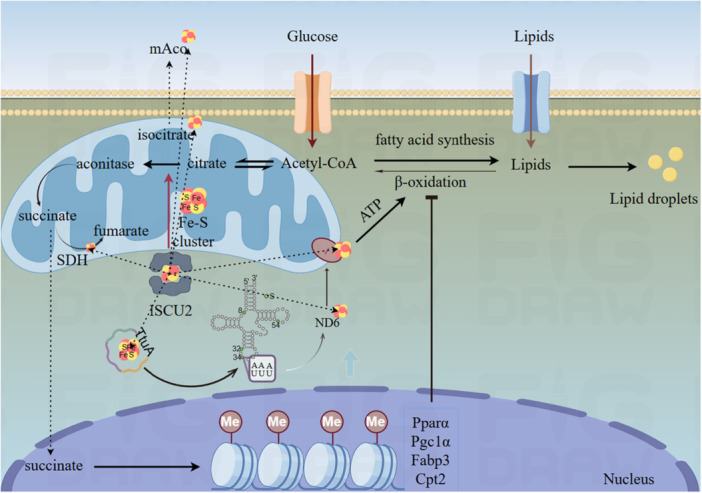
Fe–S Cluster “Failure”: A chain reaction in cellular metabolism. Fe–S cluster defects contribute to severe lipid metabolism disorders through multiple synergistic mechanisms. First, if its synthesis is impaired, it mediates the inactivation of key enzymes like aconitase and succinate dehydrogenase, leading to citrate accumulation, which in turn redirects metabolic carbon flow from energy production to fatty acid synthesis and stores it in the form of lipid droplets. Second, succinate dehydrogenase inactivation leads to succinate accumulation; excess succinate inhibits histone demethylases, stimulates genome‐wide DNA hypermethylation, and inhibits fatty acid oxidation. Furthermore, Fe–S clusters are responsible for tRNA thiolation modification to maintain translation fidelity; their defects lead to failure of mitochondrial tRNALys modification, mistranslation of respiratory chain Complex I subunit ND6, and failure of Complex I assembly. This not only exacerbates the energy crisis caused by reduced ATP synthesis and the inhibition of fatty acid oxidation but also significantly increases ROS production due to “electrical leakage” in the electron transport chain. Ultimately, the combined effects of energy crisis, enhanced de novo fat synthesis, blocked fatty acid oxidation, and severe oxidative stress form a “lipotoxicity‐oxidative stress” vicious cycle, ultimately leading to severe intracellular lipid accumulation and related metabolic diseases.

In the context of alcohol‐driven injury, ethanol‐mediated depletion of Fe–S clusters likely acts as a primary trigger for this translational collapse. By compromising the structural integrity of Fe–S‐dependent thiolation enzymes, ethanol predisposes the liver to ND6 mistranslation, mitochondrial respiratory failure, and the subsequent metabolic reprogramming that drives progression from simple steatosis to advanced hepatic injury (Figure 5).

### 2.4 Dysregulation of Hepatic Lipid Metabolism Driven by Fe–S Cluster Imbalance

The metabolic processing of alcohol significantly disrupts hepatic redox homeostasis, characterized by an elevated NADH/NAD^+^ ratio and consequent mitochondrial reductive stress. Central to this metabolic derangement is the impairment of Fe–S cluster‐dependent enzymes, which serves as a critical junction in the progression of Alcohol‐related Liver Disease (ALD) [83, 84].

Fe–S cluster deficiency exacerbates hepatic steatosis through a multifaceted mechanism. On one hand, it promotes citrate efflux and the generation of Acetyl‐CoA, thereby upregulating key enzymes of de novo lipogenesis (DNL), such as ACC and FAS [85, 86]. On the other hand, the activity of Fe–S cluster‐dependent enzymes involved in fatty acid β‐oxidation is severely compromised, leading to a marked reduction in lipid catabolism [87, 88]. This “increased synthesis, decreased oxidation” imbalance, compounded by ethanol‐induced reductive stress, results in the hallmark centrilobular triglyceride accumulation observed in alcoholic fatty liver [89].

As the liver attempts to compensate, it increases the synthesis of Very Low‐Density Lipoprotein (VLDL) to export excess lipids. However, in the context of persistent Fe–S cluster dysfunction, this export mechanism becomes insufficient, leading to intrahepatic lipid retention and secondary lipotoxicity [90]. The excessive accumulation of lipids, particularly free fatty acids (FFAs), triggers a secondary wave of injury: First, oxidative and ER Stress: Lipid overload enhances the activity of CYP2E1 and generates massive Reactive Oxygen Species (ROS), which directly attack hepatic membranes and proteins [91]. Second, inflammatory Cascade: ROS and lipid peroxides activate the TLR4/NF‐κB signaling pathway within hepatocytes and Kupffer cells, promoting the secretion of pro‐inflammatory cytokines (e.g., TNF‐α, IL‐6) [92]. Third, progression to Steatohepatitis: This transition from simple steatosis to alcoholic steatohepatitis (ASH) is further accelerated by the loss of Fe–S cluster‐mediated antioxidant defenses, which renders hepatocytes vulnerable to apoptosis and fuels the activation of hepatic stellate cells, the precursors to liver fibrosis [93, 94].

In summary, the loss of Fe–S clusters does not merely cause transient lipid accumulation but acts as a metabolic “tipping point” that drives the liver from manageable metabolic stress toward chronic inflammation and irreversible structural damage. By focusing on the intrahepatic consequences of Fe–S cluster imbalance, we can better understand the evidence‐based progression of ethanol‐driven hepatic metabolic injury.

## 3 Targeting Fe–S Clusters and Lipotoxicity: A Novel Therapeutic Perspective for ALD

Iron‑sulfur cluster protection strategies represent a critical direction for the prevention and treatment of alcoholic liver disease (ALD). Given the direct damage of ROS to Fe–S clusters, the application of antioxidants is essential [95]. For instance, endoplasmic reticulum‑targeted CYP2E1 inhibitors (e.g., vitamin E nanoemulsions) can alleviate oxidative stress in hepatocytes and reverse ALD progression [96]. Moreover, Nrf2 activators enhance cellular antioxidant defense capacity by upregulating antioxidant enzymes [97], while mitochondria‑targeted antioxidants such as MitoQ directly act on mitochondria to reduce ROS generation, thereby preserving the integrity of Fe–S clusters^9^. Iron overload exacerbates ferroptosis and lipid peroxidation in ALD [98], which can be effectively mitigated by regulating iron homeostasis. Iron chelators (e.g., deferiprone) selectively scavenge free iron and inhibit the Fenton reaction to reduce hydroxyl radical production. Overexpression of frataxin has been proven to reverse alcohol‑induced iron metabolic disorders and attenuate hepatocyte ferroptosis [19]. Meanwhile, as sulfur atoms are essential components of Fe–S clusters [99], supplementation with sulfur donors, for instance L‑cysteine, may facilitate the synthesis and stabilization of Fe–S clusters.

Lipotoxicity intervention strategies are also core components of ALD management. Improving fatty acid metabolism is a key measure: PPARα agonists (e.g., fenofibrate) restore fatty acid oxidation and reduce hepatic lipid accumulation, whereas FXR agonists (e.g., obeticholic acid) suppress SREBP‑1c to de novo fatty acid synthesis. AMPK activators (e.g., metformin) boost cellular autophagy and promote lipid droplet clearance [100], alleviating lipotoxic injury via enhanced lipophagy. For cell membrane protection, vitamin E supplementation strengthens the anti‑peroxidative capacity of hepatocytes and shields cell membranes from lipid peroxidation damage [101]. Since ethanol exposure disrupts vitamin E metabolism, targeted supplementation or modulation of its metabolic pathways is vital for counteracting alcohol‑induced lipotoxicity. Ferroptosis inhibitors including vitamin E, vitamin K and ubiquinone directly inhibit phospholipid peroxidation, mitigating alcohol‑mediated lipotoxic effects and cell death [19]. Additionally, targeted and untargeted lipidomic approaches enable in‑depth exploration of ethanol‑induced alterations in lipid mediators and membrane protective pathways, providing evidence for novel therapeutic target development. Combined strategies integrating Fe–S cluster protection, lipotoxicity intervention and membrane protection via vitamin E offer more comprehensive and effective approaches for ALD prevention and treatment [102]. Subsequent investigations should continue to validate the efficacy of these therapeutic and hepatoprotective agents in the treatment of ALD. In addition, further research is needed to uncover molecular mechanisms and develop drugs that target Fe–S clusters and their redox dynamics during ethanol exposure.

## 4 Conclusion

**Figure 6:**
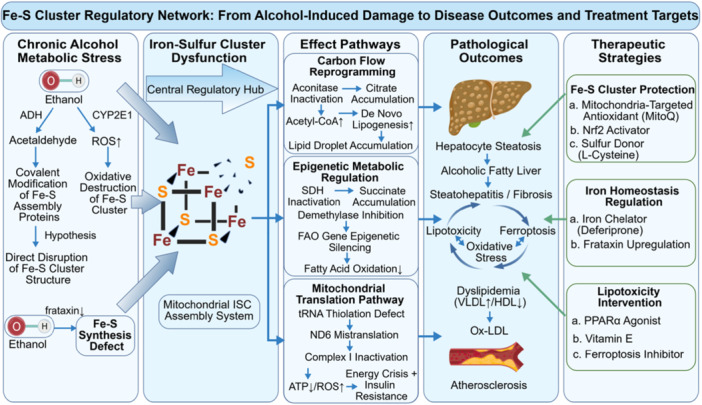
Central Fe–S cluster regulatory axis bridging chronic alcohol metabolic toxicity, multi‐organ pathological sequelae, and targeted therapeutic modalities. This schematic delineates the hierarchical pathogenic cascade initiated by chronic ethanol metabolic stress, with mitochondrial Fe–S cluster dysfunction serving as the indispensable central regulatory node. Ethanol exerts multi‐layered inhibitory effects on Fe–S cluster integrity and biogenesis: acetaldehyde produced by ADH‐mediated ethanol oxidation covalently modifies core Fe–S assembly machinery to disassemble mature Fe–S clusters. CYP2E1‐driven ROS overproduction induces oxidative destruction of Fe–S prosthetic groups. Ethanol‐dependent frataxin downregulation cripples the mitochondrial ISC assembly system, resulting in global defects in Fe–S cluster synthesis. Functional loss of Fe–S‐containing enzymes disturbs three interconnected signaling branches: Carbon flux reprogramming: aconitase inactivation causes citrate accumulation, elevated acetyl‐CoA, and excessive de novo lipogenesis, leading to massive hepatic lipid droplet deposition. Secondly, epigenetic‐metabolic crosstalk: SDH inhibition triggers succinate buildup, suppresses histone demethylase activity, epigenetically silences FAO genes, and blunts hepatic lipid catabolism. Finally, mitochondrial translational failure: impaired tRNA thiolation causes ND6 mistranslation, respiratory complex I dysfunction, ATP depletion, and amplified ROS production, culminating in energy deficit and peripheral insulin resistance. Collectively, these Fe–S‐dependent pathways drive progressive alcoholic liver disease (from simple steatosis to steatohepatitis and liver fibrosis), and establish a self‐amplifying pathological loop among lipotoxicity, oxidative stress, and ferroptosis. Systemic dyslipidemia (increased VLDL, decreased HDL, elevated Ox‐LDL) further propagates atherosclerotic vascular lesions. Three distinct therapeutic frameworks are proposed to counter alcohol‐induced tissue injury: mitochondrial‐targeted Fe–S cluster protective agents, iron homeostatic regulators to mitigate labile iron overload, and pharmacological interventions to suppress lipotoxicity and ferroptosis, with exemplary small‐molecule compounds listed for each therapeutic category.

Fe–‐S clusters, as highly conserved inorganic cofactors in organisms, extensively participate in core biological processes including electron transport, enzyme activity regulation, gene expression control, and DNA repair by virtue of their characteristic topological structures. Their biosynthesis is mainly mediated by the mitochondrial ISC system, and this assembly process is susceptible to interference from factors such as genetic variation and oxidative stress. Once their function is impaired, they can induce lipid metabolism disorders through multiple regulatory pathways, forming an emerging integrative framework of “Fe–S cluster dysfunction ‐ metabolic disorder ‐ disease occurrence”. Among these, ROS and acetaldehyde generated by chronic alcohol metabolic stress damage the structure of Fe–S clusters through oxidative damage and potential covalent modification pathways, respectively, leading to intracellular iron metabolism imbalance and ferroptosis progression. Fe–S cluster damage drives lipid metabolism disorders through three key mechanisms: inducing aconitase inactivation, reducing succinate dehydrogenase activity, and disrupting tRNA thiolation modification. Ultimately, it causes systemic lipid metabolism disorders including abnormal hepatic lipid accumulation, vascular endothelial injury, and atherosclerosis, serving as a crucial regulatory hub for the occurrence and development of metabolic diseases (Table 1). This central regulatory hub also opens up promising translational therapeutic avenues: targeting iron homeostasis with iron chelators (e.g., deferiprone) can mitigate free iron overload and Fenton reaction‐induced Fe–S cluster degradation, mitochondria‐targeted antioxidants (e.g., MitoQ) can preserve mitochondrial redox balance and maintain Fe–S cluster biogenesis, and ferroptosis inhibitors (e.g., vitamin E, ferrostatin‐1) can interrupt the vicious cycle between Fe–S cluster damage and lipid peroxidation (Figure 6). These mechanism‐based targeted strategies represent key future research directions for alcoholic liver disease and related metabolic disorders.

**Table 1:** Evidence classification for mechanistic links between ethanol‐induced Fe–S cluster dysfunction and metabolic disorders.

Proposed mechanistic link	Evidence type
Chronic ethanol metabolism → ROS/acetaldehyde overproduction → Fe–S cluster structural damage & biosynthesis inhibition	Direct
Fe–S cluster deficiency → Aconitase inactivation → Citrate accumulation → Carbon flux redirection → De novo lipogenesis & lipid droplet formation	Direct
Fe–S cluster damage → Succinate dehydrogenase (SDH) inactivation → Succinate accumulation → Epigenetic inhibition → Fatty acid oxidation (FAO) suppression	Indirect
Fe–S cluster defect → TtuA‐mediated tRNA thiolation impairment → Mitochondrial translational infidelity → Respiratory complex I dysfunction → Energy crisis & ROS surge	Direct
Combined metabolic disorders → Hepatocyte lipotoxicity & hepatic steatosis	Direct
Hepatic lipid overload → Elevated VLDL & reduced HDL → Systemic dyslipidemia	Indirect
Acetaldehyde electrophilic attack → Fe‐C bond formation → Fe–S cluster distortion & disintegration	Hypothetical

This table illustrates the Fe–S cluster‐centered regulatory network in ethanol‐related metabolic injuries. Mechanisms are classified as direct experimental evidence (validated in cells, animals, and clinical samples), indirect supportive evidence, and the author's hypotheses.

## Author Contributions

**Li Xu:** conceptualization, writing – review and editing, funding acquisition. **Zhenyang Xu:** conceptualization, writing – original draft, visualization. **Chenli Su:** conceptualization, methodology. **Yanqian Zhao:** conceptualization, methodology. **Linjing Qiang:** methodology, conceptualization. **Xiayong Jing:** writing – review and editing, supervision. **Yuxiang Sun:** funding acquisition, supervision.

## Ethics Statement

Ethical statement is not applicable for this article.

## Consent

The authors have nothing to report.

## Conflicts of Interest

The authors declare no conflicts of interest.

## Data Availability

The authors have nothing to report.
